# Do patient safety incident investigations align with systems thinking? An analysis of contributing factors and recommendations

**DOI:** 10.1136/bmjqs-2025-019063

**Published:** 2025-09-12

**Authors:** Lorelle Bowditch, Charlotte Molloy, Brandon King, Masoumeh Abedi, Samantha Jackson, Mia Bierbaum, Yinghua Yu, Louise Raggett, Paul Salmon, Jeffrey Braithwaite, Johanna I Westbrook, Robyn Clay-Williams, Raghu Lingam, Sandy Middleton, Farah Magrabi, Virginia Mumford, Peter Hibbert

**Affiliations:** 1 Macquarie University Australian Institute of Health Innovation, Sydney, New South Wales, Australia; 2 IIMPACT in Health, Allied Health and Human Performance, University of South Australia, Adelaide, South Australia, Australia; 3 University of the Sunshine Coast Centre for Human Factors and Sociotechnical Systems, Sippy Downs, Queensland, Australia; 4 Nomic Research, North Turramurra, Sydney, New South Wales, Australia; 5 UNSW School of Clinical Medicine, Sydney, New South Wales, Australia; 6 Nursing Research Institute - St Vincent’s Health Network Sydney & St Vincent’s Hospital, Melbourne, Victoria, Australia; 7 Australian Catholic University School of Nursing Midwifery and Paramedicine Melbourne Campus, Sydney, New South Wales, Australia

**Keywords:** Patient Safety, Incident reporting, Human factors, Significant event analysis, critical incident review

## Abstract

**Background:**

Globally, up to 17% of hospitalised people suffer a patient safety incident. Learning from adverse events through patient safety investigation is critical to prevention; however, their utility is still questioned. Two key investigation outputs include identifying contributing factors (CFs) and proposing recommendations to prevent future occurrences. Criticisms of current methods include incomplete analysis of CFs and weak incident prevention strategies. A proposed solution is systems thinking analysis, which recognises healthcare complexity. However, it is not clear whether such methods are being applied in practice.

**Objective:**

This study aimed to assess current use of systems thinking-based strategies by examining a set of Australian patient safety incident investigations.

**Methods:**

Investigations (n=300) from 56 different Australian health services were deductively analysed. Identified CFs were classified by healthcare system level using a framework combining Systems Engineering Initiative for Patient Safety (SEIPS) principles and AcciMap’s hierarchical structure. Recommendation sustainability and effectiveness were classified as weak, medium or strong using US Department of Veteran Affairs’ criteria.

**Results:**

51% of incidents were issues with clinical processes and procedures. The investigations identified CFs that disproportionally focused on the people involved in those processes (n=677, 47%) rather than other system levels and as a consequence, most recommendations were of medium (n=665, 51%) and weak (n=560, 43%) strength. Notably, 10% of investigations lacked any CFs or recommendations.

**Conclusion:**

The focus on individual actions highlighted that simple linear thinking persists in patient safety incident investigations. This study proposes five key areas of effective incident analysis and investigation: a sociotechnical focus; improved data collection techniques; investigative independence; the professionalisation of investigators; and the aggregation of data. Learning from incidents is key to maximising their preventative effectiveness, especially in an increasingly complex healthcare system.

**WHAT IS ALREADY KNOWN ON THIS TOPIC**

- Contemporary high-income health systems generally deliver high-quality care; however, patients continue to experience preventable harm. Many healthcare systems seek to reduce these events through analysis and investigation, identifying contributing factors and developing recommendations.

**WHAT THIS STUDY ADDS**

- This study demonstrates a clear research–practice gap in healthcare around incident investigation and analysis methods. The contributing factors identified in incident investigations are predominately person-focused and recommendations are relatively weak, despite an evident attempt at being systems-focused.

**HOW THIS STUDY MIGHT AFFECT RESEARCH, PRACTICE AND POLICY**

- Incident investigations are limited by explicit and implicit constraints such as resourcing, investigator training and methodological challenges which require attention in order to improve their effectiveness.

## Introduction

Contemporary high-income health systems generally deliver high-quality care; however, patients continue to experience preventable harm.^1 2^ Worldwide, the proportion of hospitalised patients who experience a patient safety incident during their care ranges between 4% and 17%,^3 4^ many of which were preventable.^5^ Preventable patient safety incidents, sometimes referred to as adverse events, account for over US$600 billion per year in health expenditure worldwide according to Organisation for Economic Co-operation and Development (OECD) estimates.^5^

Incident analysis and investigation are key tactics within a broader strategy employed by many healthcare systems to reduce the unacceptable burden of adverse events. Investigations of serious harm incidents aim to understand their underlying causes, with key outputs including identification of contributing factors (CFs) (also known as contributory factors^6^) and recommendations for incident prevention strategies.^2^ In contemporary safety science, adverse events represent a complex systems phenomenon.^7^ Sociotechnical systems recognise the interaction between people and technology in workplaces and how they influence outcomes.^8^ Adverse events arise from interactions of CFs at all levels of sociotechnical systems.^9^ In healthcare, this means that to best understand incident causation, a multilevel, systems thinking view that recognises the interdependencies between system components is required.^10 11^ The increasing recognition of healthcare complexity means that a focus solely on CFs associated with the individuals closest to an incident will overlook systemic weaknesses at different levels of the healthcare system including broader local, organisational or national influences, and even societal trends.^12 13^ The identification of CFs typically informs a set of recommendations designed to be implemented to prevent reoccurrence of similar incidents impacting other patients and to enable organisational learning.^14^ These recommendations can be variably effective and sustainable in improving care to patients.^15 16^

Even with concerted efforts in the preceding decade,^17^ there is little evidence that incident analysis and investigation methods in healthcare are associated with reductions in patient harm.^2 18 19^ This is despite an analysis and investigation’s role in providing the context for identifying CFs, which are an essential precursor for generating and implementing recommendations aimed at improving outcomes. A recurring criticism is that there is a lack of a systems thinking approach, both in the methods applied and the philosophy underpinning investigations.^17 19^ As a result, CFs and recommendations are focused too heavily on changing the decision-making, behaviours and knowledge of clinical staff; and less focused on the macro-environment in which clinicians work and the broad societal influences to which they are subjected.^20 21^ Previous studies have shown that recommendations tend to be weak (less likely to be effective and sustainable), largely targeting knowledge gaps that clinicians are purported to have.^15 22^ Strong (more likely to be effective and sustainable) and medium (possibly effective and sustainable) strength recommendations are less likely to be suggested—meaning that incident analysis is unlikely to inform practice or process improvements.^16^

Despite repeated calls for a systems thinking approach to analysis and investigation,^23 24^ it is not clear whether such methods are being applied in practice. This study aimed to address this evidence gap by evaluating Australian patient safety incident investigations (sometimes referred to as ‘reviews’) to examine the extent to which a systems thinking perspective was adopted.

## Methods

This study employed a directed qualitative content analysis design.^25^,^28^ This approach has been previously used effectively in patient safety research.^26^,^28^ Directed content analysis incorporates previously established theory to guide a focused, deductive analysis.^25^

### Study setting

Public health systems in three Australian states—New South Wales (NSW), Victoria and Queensland (QLD)—contributed to this study. These states account for over 21 million Australians—more than 77% of the population of Australia.^29^ Safer Care Victoria, Clinical Excellence Commission (NSW) and Clinical Excellence Queensland provided investigation reports to the research team. Health services undertake investigations and share them with these state-based quality and safety organisations. Investigation reports are undertaken at a multitude of healthcare settings, such as acute hospitals, community and mental health services and ambulance services but not general practice, which are dealt with separately. A complete list of incident investigations rated at the highest level of harm (NSW Harm score 1,^30^ Queensland severity assessment code 1,^31^ Victoria incident severity rating 1^32^) from 2022 and 2023 was requested from each state. From this, reports were excluded based on unavailability at state level, that is, restricted under legal protection in QLD, certain review types (e.g., COVID-19 rapid reviews) in NSW and investigations deemed ‘high public interest’ in Victoria. The investigations were then randomly sampled, stratified by incident type and investigation type, to obtain a sample of 100 reports per state (online supplemental figure). This process ensured a representative sample of the types of serious patient safety incidents and investigation types in Australia and was consistent with established principles for achieving proportional representation of subgroups within a larger population.^33 34^

### Analysis

Individual investigations were categorised using a four-step, deductive classification process, using pre-existing and adapted frameworks which are described in detail below. In step 1, each incident investigation report was read in detail and the WHO’s International Classification for Patient Safety (ICPS) framework^35 36^ was used to identify the primary incident type. This step did not involve the same complex deductive interpretation as the latter steps. It was therefore conducted as a separate analysis by a single researcher under the direction of a patient safety expert to ensure consistency and accuracy, in line with patient safety research.^37^

Five researchers completed steps 2–4. In step 2, demographic information including the jurisdiction, the incident analysis method, the admitting specialty and patient characteristics was also extracted from each report. Steps 3 and 4 are outlined in detail in the following sections.

#### Analysis of CFs

Step 3 involved extracting the wording of each CF (explicitly implicated in the incident and labelled as contributing/contributory factors or findings) and Lessons Learnt (influencing factors that were identified but did not directly contribute to the event and were labelled as lessons learnt or as unrelated to causal or contributing/contributory factors) from every investigation report. Extracted CFs and Lessons Learnt were combined into a single dataset labelled ‘CFs’. Lessons learnt were included to ensure all considered systemic issues were captured. Each CF was deductively classified using a framework that combined elements from both AcciMap^38^ and Systems Engineering Initiative for Patient Safety (SEIPS)^39^ models. The framework, based on principles of systems thinking, was developed by the research team and allowed for the CFs to be classified to a sociotechnical system level. AcciMap provided the structure for the hierarchical levels and SEIPS provided categories to classify the CFs within the levels. To ensure a hierarchically appropriate classification framework that could address the broad range of internal and external factors that could be identified in the CFs, SEIPS 2.0^40^ and 3.0^41^ were used as a foundation for separating the physical (internal) and external environment. These more recent versions of SEIPS reflect a sociotechnical systems approach to the patient journey^41^ and posit that six elements make up any work situation: the people; the tasks; the tools; the physical environment; the organisation; and the external environment.^42^ The term ‘physical’ when referencing the environment is referenced as ‘internal’ in these models and is used in the current study to reflect the more local surroundings directly controlled and experienced within the healthcare setting. This label better suited the lower system levels of the AcciMap hierarchy and made it distinct from the broader external environment captured in the higher levels.^43^

Each CF was read in detail and was assigned to one or more of the predefined system levels and CF classification within the framework. The complete framework can be found in the online supplemental table; however, the following is a list of the system-level categories with examples and the CF classifications:

1. External environment: government budgets, social and cultural influences.
2. Organisation: facilities management and maintenance, organisational directives and policies.
3. Person: care team communication, the patient’s clinical condition.
4. Tasks: task difficulty, job demand versus available capacity.
5. Tools and technology: ease of use of tools, local protocols and practices.
6. Internal environment: the location of the workspace, noise.

#### Analysis of recommendations

In step 4, like with the CFs, the wording of each recommendation (changes/improvements proposed in the reports in response to the findings) was extracted from every investigation report. Recommendations were then read in detail and deductively classified using an adapted version of the US Department of Veteran Affairs’ recommendations effectiveness criteria.^16 44 45^ This framework is designed to classify the type and strength of recommendations, and in turn, represents their likely effectiveness and sustainability.^16^ The strength of recommendations was presented as ‘strong,’ ‘medium’ or ‘weak’.^15 44 45^ Strong recommendations (e.g., engineering controls) rely less on changing the actions of people and are more likely to be effective and sustainable. Medium recommendations (e.g., standardisation of communication tools) are more related to changes in current policy, guidelines and practices, whereas weak recommendations (e.g., training and education) rely on changes to human behaviour and are less likely to lead to effective and sustainable change.^16 44^ The following is a list of each of the strength categories including healthcare examples:

1. Strong: alter the electronic medical record form so that alterations to the medical emergency or urgent clinical review call criteria must be authorised by registrar level or above.
2. Medium: review of the Safety Huddles programme to ensure they are structured and increase the opportunity to identify safety risks.
3. Weak: ensure that learnings are shared with the relevant department.

Classifications of both CFs and recommendations then underwent descriptive analysis.

### Quality assurance and inter-rater reliability

Initial ICPS classification was completed by two researchers (C1, C2) who jointly classified 20 investigations. A further 40 were independently classified by both researchers to determine agreement. All remaining ICPS classification was conducted by C1. This is consistent with the approach used in patient safety research.^37^ Inter-rater reliability was assessed using Cohen’s Kappa. Cohen’s Kappa for the primary incident type was 0.95 (95% CI, 0.884 to 1.02), p<0.001, indicating almost perfect agreement.^46^ There was also almost perfect agreement at level 2 of the primary incident (κ=0.82 (95% CI, 0.711 to 0.934), p<0.001) which classified the incident process or ‘what went wrong’.

For classifying CFs and recommendations, five researchers were assigned a varying proportion of the dataset (ranging from 33 to 80 investigations). Each report was analysed by a single researcher. However, different groups of researchers worked on the classification task at different time points, with a maximum of two researchers working concurrently at any one time.

To ensure consistency and validity of the coding, weekly inter-rater reliability discussions were conducted among these groups, which were facilitated by an expert in patient safety each time. These meetings served as a forum for achieving consensus on ambiguous or unclear classifications. An example of a point of discussion came from needing to clarify how to distinguish between organisation policies and procedures from more local protocols and practices.

The coding process was guided by a set of mutually agreed rules in the form of a codebook, which was version-controlled. This codebook contained rules and definitions for classifying CFs and recommendations. Changes that were decided during the weekly meetings were documented in the codebook. All changes to the codebook, including new rules, definitions and classification names, were documented in a new version. An example of how the codebook enhanced the reliability of the coding can be found in a new classification that was developed for recommendations related to ‘sharing learning’. Following its inclusion, after agreement that it should be distinguished from other classifications, all relevant references to sharing learning were recoded to reflect the addition. This approach to inter-rater reliability and the iterative codebook ensures the validity and reproducibility of the classification system. The research, specifically framework development, was conducted in collaboration with experts in patient safety and human factors to adhere to rigorous methodological standards.

## Results

### Patient safety incident investigation characteristics

Three hundred incident investigations from 56 different Australian health services across three states were reviewed. Table 1 demonstrates the structure and format of the incident investigation reports. QLD had the most variability in their templates with 18 different formats. Conversely, Victoria used one consistent template format yet included supplementary material with each investigation which included flowcharts, different types of causal diagrams and additional analysis. The average incident investigation report was 21.5 pages (SD=14.2, range 3–62). Reports included open text boxes for detailed narratives and summaries. Checkboxes were used for specific prompts, and investigators were guided by structured text boxes and tables for documenting CFs and recommendations. Additionally, images were sometimes used in QLD reports to support case descriptions.

**Table 1:** Incident investigation report characteristics

Design feature(s)	State		
	NSW	VIC	QLD
Template versions	5	1	18
Open text narrative	✓	✓	✓
Checkboxes	✓		✓
Structured text boxes	✓	✓	✓
Structured tables	✓		✓
Supplementary material		✓	✓
Images			✓

NSW, New South Wales; QLD, Queensland; VIC, Victoria.

General incident characteristics are shown in table 2. Most incidents occurred in principal referral hospitals (n=98, 33%) in major cities (n=190, 63%). One-third of incidents occurred on general wards or a patient’s room (n=101, 34%), followed by emergency departments (n=46, 15%). Patient age ranged from 0 to 80+ years, with 43% (n=135) of patients over 60 years of age.

**Table 2:** Characteristics of the incident investigations

Incident characteristics	N (%)
**Jurisdiction**	300
Victoria	100 (33)
New South Wales	100 (33)
Queensland	100 (33)
**Remoteness area** ^86^	300
Major city	190 (63)
Inner regional	64 (21)
Outer regional	33 (11)
Remote, very remote	6 (2)
Unknown	7 (2)
**Hospital peer grouping** ^87^	300
Principal referral hospitals	98 (33)
Public acute group A hospitals	80 (27)
Public acute group B hospitals	29 (10)
Public acute group C hospitals	23 (8)
Public acute group D hospitals	10 (3)
Women’s and children’s hospitals	10 (3)
Other†	8 (3)
NA‡	26 (9)
Unknown§	16 (5)
**Incident location**	300
General ward/patient’s room	101 (34)
Emergency department	46 (15)
Birthing suite/labour room	23 (8)
Operating theatre	22 (7)
Multiple sites	21 (7)
Outpatient clinic	9 (3)
Diagnostic procedures	5 (2)
Mental health/psychiatric unit	3 (1)
Transfer between hospital or units	2 (1)
Neonatal or paediatric ICU	1 (<1)
Nursery	1 (<1)
Coronary care or acute care unit	1 (<1)
Other (eg, in the community)	39 (13)
Not known	11 (4)
**Patient sex¶**	306
Male	160 (52)
Female	140 (46)
Unknown	6 (2)
**Patient age range§**	306
0–4 years	10 (3)
5–19 years	12 (4)
20–29 years	23 (8)
30–34 years	27 (9)
40–49 years	27 (9)
50–59 years	21 (7)
60–69 years	35 (11)
70–79 years	47 (15)
80+ years	53 (17)
Unknown	51 (17)

† Includes ‘public rehabilitation hospitals’, ‘mixed subacute and non-acute hospitals’, ‘mixed day procedure hospitals’ and ‘very small hospitals’.

‡ Includes non-hospital settings that do not have a peer grouping, such as aged care facilities, community etc.

§ Data for health service level only—therefore peer grouping unknown.

¶ Includes additional frequencies to account for multi-incident reviews (n=6) which included more than one patient.

As shown in table 3, 51% of the patient safety incident types were related to the clinical process/procedure (n=152, 51%). Within this category, problems with diagnosis/assessment (n=53, 18%) were the most common incident types, followed by procedure/treatment/intervention (n=43, 14%) issues. Together, patient falls (n=45, 15%) and issues related to patient or staff behaviour (n=31, 10%) accounted for one-quarter of the patient safety incidents.

**Table 3:** Primary incident type categories represented in the incident investigations

ICPS categories	N (%)
Clinical process/procedure	152 (51)
Diagnosis/assessment	53 (18)
Procedure/treatment/intervention	43 (14)
Deterioration	22 (7)
Stillbirth	10 (3)
Specimens/results	8 (3)
Tests/investigations	7 (2)
General care/management	6 (2)
Pressure ulcer	2 (1)
Detention/restraint	1 (<1)
Falls	45 (15)
Behaviour	31 (10)
Medication/intravenous fluids	19 (6)
Clinical administration	17 (6)
Appointment	8 (3)
Transfer of care	4 (1)
Referral/consultation	3 (1)
Handover	1 (<1)
Waiting list	1 (<1)
Healthcare-associated infection	10 (3)
Outcome not related to healthcare management	9 (3)
Medical device/equipment	8 (3)
Resources/organisational management	4 (1)
Blood/blood products	2 (1)
Patient accidents	2 (1)
Nutrition	1 (<1)

Note: only clinical process/procedure and clinical administration show level 2 of the primary incident type as they included the most variation.

### Investigation method

The most frequently used investigation method was Root Cause Analysis (RCA or RCA^2 44 45^) (n=117, 39%). London Protocol^47^,^49^ and Human Error and Patient Safety (HEAPS)^31^ analysis accounted for a further 16% (n=47) and 15% (n=45), respectively. AcciMap^38^ was used as a methodological approach in 1% (n=4) of incidents. ‘Other’ methodologies made up a large proportion of incident investigations (n=86, 29%) and frequently included Concise or Comprehensive Incident Analysis.^50^

### Contributing factors

Of the 300 incident investigations, 923 CFs were identified with a mean of 3.1 CFs per investigation (median=2.00, IQR=3.00, range 0–14). In total, 23% (n=70) of investigations identified no CFs and 43% (n=130) identified one to three CFs.

**Figure 1:**
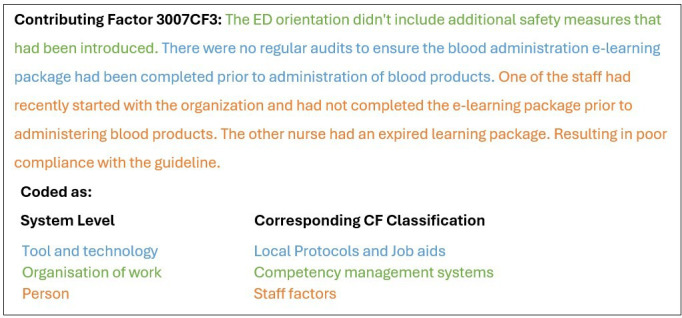
An example of a single contributing factor that included multiple system-level classifications.

Many CFs included references to multiple levels in the sociotechnical system (see figure 1 for an example). Therefore, while 923 CFs were identified in the investigation reports, the total number of classifications was 1458.

**Figure 2:**
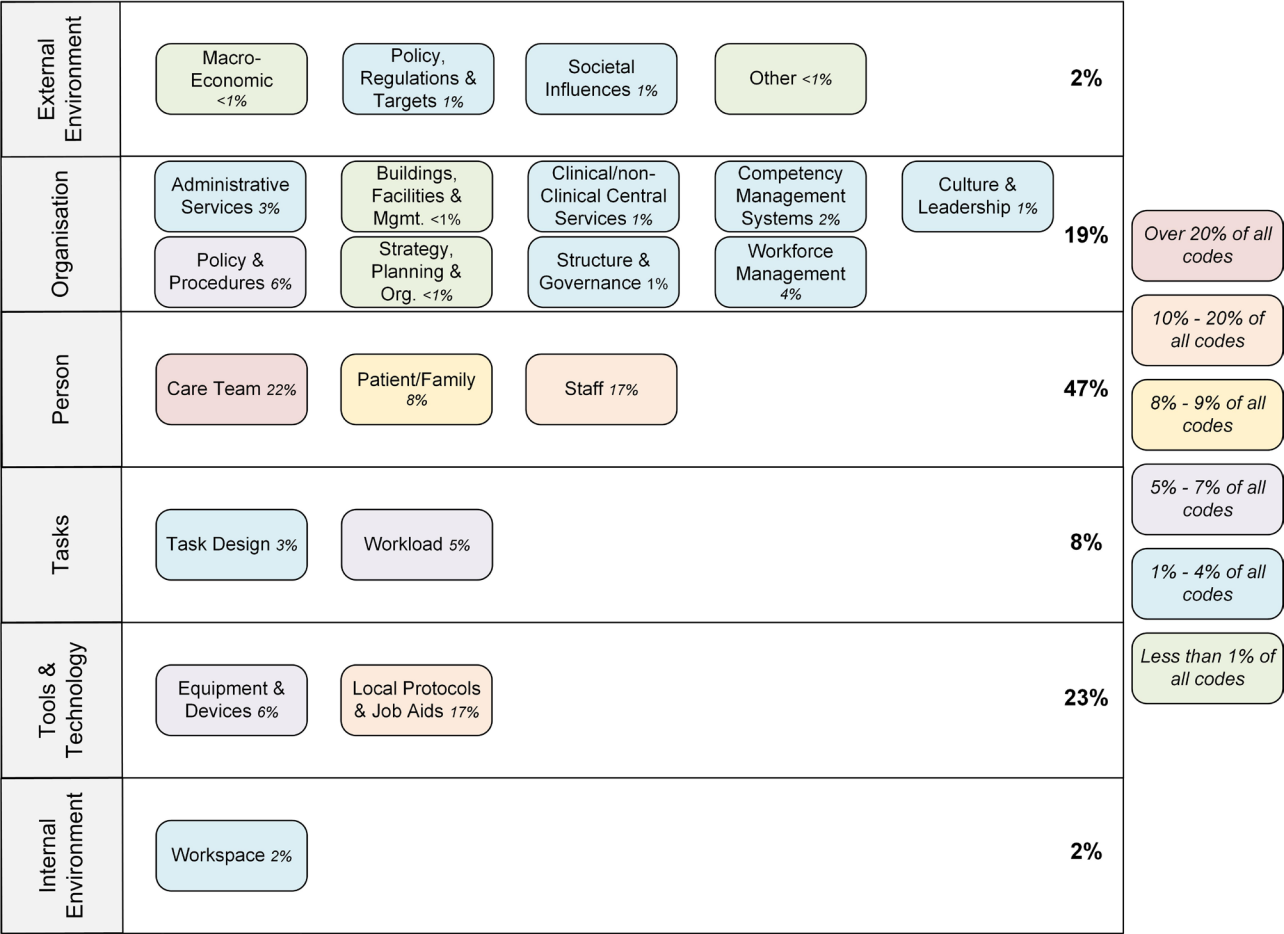
Hierarchical diagram showing contributing factors classifications at the system level, colour-coded to reflect their frequency percentage.

Figure 2 shows the frequency of these classifications, and their corresponding sociotechnical level in the system. These data are presented in a format informed by AcciMap/SEIPS, which emphasises the hierarchical structure of the system; however, it should not be interpreted as an AcciMap. Almost half of all CF classifications were at the person-level (n=677, 47%). Most frequently, these included classifications related to the care team (n=317, 22%) (e.g., communication within teams) and staff factors (n=249, 17%) (e.g., staff decision-making). The next most frequent classifications were at the tools and technology level (n=333, 23%), most often related to local protocols and job aids (n=245, 17%) (e.g., diagnosis and decision-making tools). External (n=34, 2%) (e.g., environmental conditions) and internal environment (n=35, 2%) (e.g., bed layout) system level factors were the least frequently identified.

Further analysis showed that 17% (n=51) of the 300 individual reports identified CFs at a single system level only (e.g., a report identified three CFs, but all three only included task-level classifications). Within these 51 investigations, the largest proportion (n=32, 11%) included only person-level CF classifications. Therefore, 32 individual investigations attributed the cause of the incident to *only* the people involved.

### Recommendations

There was an average of 3.4 recommendations for each incident investigation (Median=3.00, IQR=4.00, range 0–17) for a total of 1006 recommendations. About 14% (n=42) of investigations included no recommendations (n=29 of which also included no CFs), and 44% (n=133) included one to three recommendations.

**Figure 3:**
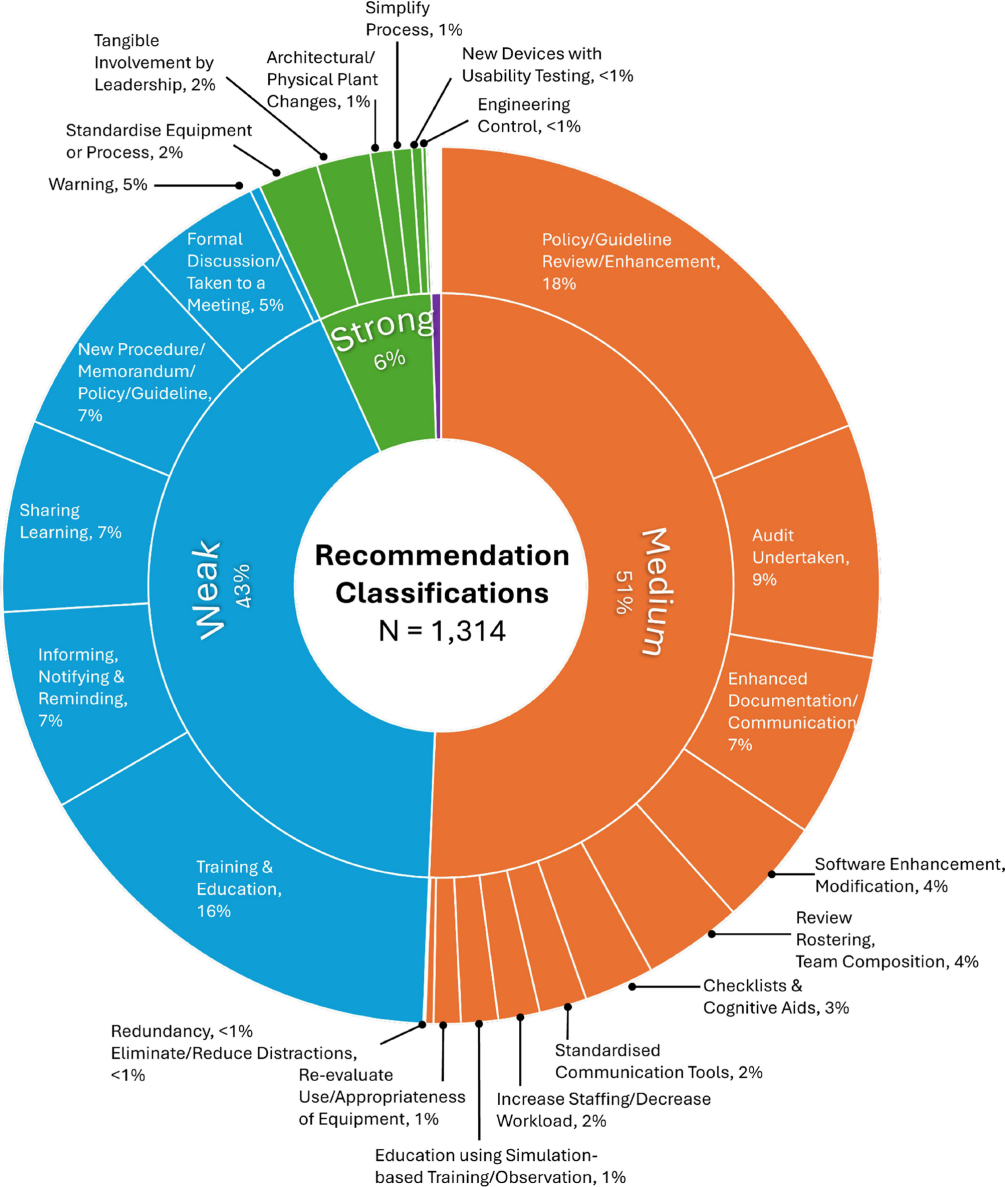
Sunburst diagram showing frequency of each recommendation classification, including ‘other/not a recommendation’ (n=7, 1%) category in purple. (Categories adapted from the US Department of Veteran Affairs’ recommendations effectiveness criteria^16 44 45^).

As with the CFs, some recommendations included multiple strength classifications (e.g., weak) and corresponding type (e.g., sharing learning). For this reason, 1314 individual strength classifications were identified. As shown in figure 3 more than half of all recommendation classifications were of medium strength (n=665, 51%), closely followed by weak strength classifications (n=560, 43%). Only 6% (n=82) of all recommendation classifications were rated as strong. The most frequent recommendation types were related to reviewing or enhancing existing policies or guidelines (medium: n=250, 18%), closely followed by training and education (including counselling) (weak: n=211, 16%). About 10% (n=30) of the 300 incident investigations proposed only weak recommendations, and 20% (n=61) of all incident investigations included at least one strong recommendation.

## Discussion

### Interpretation of the findings in the context of the wider literature

This study assessed patient safety incident investigations to determine the extent to which a systems thinking perspective has been adopted when identifying CFs and proposing recommendations. Findings revealed that the most common incident type involved clinical processes and procedures, reflecting national and international patterns of serious adverse events.^51 52^ Person-level factors, particularly those related to the care team (e.g., communication within teams), were identified in 47% of CFs, and in 11% of all investigations, *only* person-level factors were identified. This aligns with patient safety literature, where individual factors are among those most frequently recorded.^15 53 54^ These findings highlight the difficulty in moving beyond easily identifiable CFs to identify broader systemic factors, ultimately impeding organisational learning.^22^

Our findings also provide further evidence of a research–practice gap in healthcare surrounding the approach to adverse event analysis.^55^ Specifically, while it is widely acknowledged in the academic literature that investigations should be underpinned by a systems thinking approach,^56^ this is not reflected in practice where a reductionist approach focused on staff and their equipment prevails. This is evident with the low median (2.00) of CFs identified per investigation and 23% of investigations finding no CFs. In contrast, recent research into aviation incidents showed that when incident analyses are conducted by human factors experts, the number of CFs identified per investigation is often higher (15–16 CFs).^57 58^ This disparity could be linked to the focus on factors closer to the individuals involved with less consideration of the broader organisational and external factors that might have contributed to the incident but were less obvious.

The focus on the individuals and team closest to the incident—known as a ‘fixing broken components’ approach—might explain why only 6% of all recommendations were classified as strong. This is consistent with other research that generally reports strong recommendations in just 3%–8% of cases.^15 16 59^ This approach results in less effective interventions as the broader systemic conditions that influence individual behaviour are not dealt with.^43 60^ The vast majority (>90%) of recommendations were classified as medium or weak, most of which were targeting improvement of existing policies and procedures or training and education. While weak recommendations can be valuable when paired with stronger, more sustainable solutions, in isolation they have limited effectiveness and change is often difficult to sustain.^15 16 21 59^ Clearly, the challenge facing patient safety incident investigators remains in the development of strong, actionable recommendations that promote sustainable safety improvements. The adoption of a systems thinking approach during investigation is critical to support this.

The analysis of both CFs and recommendations, a perspective often overlooked by previous research, highlights a novel approach. Numerous studies offer insight into recommendations^15 16 59 61^ but fewer focus on CFs as well. It is important to examine both key outputs, as CFs should directly inform the development of recommendations. By doing so, this study sheds light on the analytical pathway that investigators use when constructing recommendations based on their CF findings. The provision of healthcare is a complex sociotechnical process,^62^ not a simple linear one.^63^ These findings suggest that when investigations focus on individual actions, incidents tend to be framed as being influenced by individual behaviours. The consequence of this is weaker, less effective recommendations.

Despite patterns that suggest a simple linear approach, almost every incident investigation stated that a systems thinking-based approach was used. A linear approach to investigation understands incidents as the culmination of a series of events,^64^ for example, A causes B which causes C.^10^ In contrast, a systems thinking-based approach is designed to capture the complexity of the healthcare system,^62^ positing that incidents arise from systemic flaws, rather than the flaws of the individuals present within the system.^65^ Healthcare, as a complex system, has interdependencies between people, teams, organisational environments, processes, equipment and technology^10 11^ as well as broader influences like regulations, international standards and society. For this reason, a systems thinking-based investigation should identify higher-level systems factors, with less focus on the individuals in the system. Despite this, only 19% of all CF classifications were at the organisational level. This, coupled with less effective and sustainable recommendations, suggests a disconnect between the intention and the application of the system thinking-based approach. One reason for this disconnect is the use of investigation methods that do not fully incorporate core systems thinking principles. In the present study, the most applied methods were RCA, the London Protocol and HEAPS—some of which are mandated in various jurisdictions (e.g., RCA and London Protocol are among the four permitted methods in NSW^30^), but have received criticism in the past.^66^ AcciMap is arguably the most applied systems thinking method across various safety critical domains^43 66^ and is among the suggested methodological approaches in one of the jurisdictions in this study (Victoria^32^). However, AcciMap was used in just 1% of all investigations.

The expertise of the investigation team may also be an issue. Incident investigations are often conducted by multidisciplinary teams dominated by clinicians^67^ who often lack human factors expertise^16 21 68^ and have a varying knowledge of safety theories and safety investigation methods.^69^ While this multidisciplinary approach is an obvious strength, offering different perspectives, it could also influence linear causal explanations rather than systemic ones, due to a lack of knowledge of complex system theories.^69^

Ramsey *et al*^70^ reported that incident investigators felt constrained to produce coherent descriptions of events despite conflicting accounts and gaps in understanding what happened. Investigators may also lack confidence that their recommendations would be implemented.^70^ This can be exacerbated by organisational influences impeding investigators (either implicitly or explicitly) from developing recommendations beyond the health service level.^71^ This can be compounded by undervalued roles and a perceived lack of action from managers who can struggle to endorse recommendations due to inadequate resourcing and budgetary demands.^72 73^

### Implications for policy, practice and research

Our findings have implications related to how patient safety incident investigations are conducted. The framing of incidents with a focus on individual actions highlights the linear thinking that persists in patient safety incident investigations. The consequence of this was that a third of all recommendations were focused on either reviewing existing policies or staff education. This pattern suggests that incident investigations are identifying human error within the prescribed clinical workflow and expecting adherence to policies and assumed knowledge, or ‘work-as-imagined’.^74^ The response, or recommendation, is an attempt to enhance these policies and knowledge.^75^ However, when work-as-imagined^76^ is used as the basis of the investigation of an adverse event, areas of key importance reflecting ‘work-as-done’, such as the habitual activities of clinicians, managers and supporting staff, can be missed.^76^

Work-as-done may not be considered because incident investigations frequently rely solely on information collected from staff interviews and reviews of documentation.^16^ This approach limits understanding, as person-level factors and workflow issues are easily identifiable in these sources, and thus remain the focus.^21^ Less often, human factors techniques such as observations^77^ and simulation^78^ are used, despite being essential to identifying the dynamic circumstances and variability of everyday practice—or work-as-done.^76^ These types of techniques are central to safety and quality improvement and should be incorporated to help to bridge the gap between work-as-imagined and work-as-done to enhance the understanding of adverse events.^76^

Broadly, there is an opportunity to learn important lessons from the best practice principles of safety-critical industries such as the aviation, maritime, railway industries^79^ and some healthcare organisations such as the Health Services Safety Investigations Body (HSSIB) in England and the Norwegian Healthcare Investigation Board (NHIB). These are dedicated, independent and permanently staffed bodies, which enables them to investigate safety risks and failures.^79^ This independence allows for consistent, system-wide investigations which are centred on learning and improvement^80^ and are not constrained by the same organisational demands as internal investigations.

AcciMap is the dominant sociotechnical system analysis approach used for investigating safety-related problems in safety critical industries.^81^ Although this approach is used in healthcare agencies,^81^ human factors and ergonomics expertise are underutilised. This expertise is a vital component of good quality analysis and outcomes.^68^ The findings of the current study present a clear need for the professionalisation of incident investigators, equipping the team with an understanding of complex sociotechnical systems methodologies to effectively lead, or at least be part of serious patient safety incident investigations.^68 79^

The ability to aggregate incident data across multiple events to learn about safety risks is developing across the healthcare system.^82^ This practice offers significant promise, where aggregation of incident data allows investigators to identify complex patterns of CFs, and acts as a powerful tool to enhance the development and implementation of interventions that improve healthcare.^7^ It is notable that analysis methods like AcciMap have been used successfully to support incident aggregation and the identification of trends in other sectors.^83^

### Strengths and limitations

This study provides insights from a large sample of patient safety incident investigations from three Australian jurisdictions. Together, the states of NSW, QLD and Victoria comprise 77% of the population of Australia^29^ and cover 77% of all hospital admissions and emergency department presentations.^14^ By exploring patterns across 300 investigations, the findings revealed insight into how system factors overall are being considered in serious incidents, offering a more generalised perspective than the examination of a single incident could provide.^31 82^ Learning from patient safety incidents is fundamental to improving patient safety.^84^ Identifying recurring patterns in CFs and the recommendations developed during the incident investigation process highlights opportunities to strengthen systems thinking and, in turn, reduce patient harm. While beyond the scope of this paper, future research could explore the comparative efficacy of different investigation methods in identifying CFs and proposing recommendations. This could include examining cooccurrence patterns both within and between CFs and recommendations.

A potential limitation of this study is the inherent subjectivity of deductive qualitative analysis.^25 85^ To reduce the potential of bias, quality assurance processes were implemented, including inter-rater reliability tests, as well as engagement in regular reciprocal reflexive conversations and well-defined frameworks and business rules to guide the analysis to which changes were documented following these conversations.

## Conclusion

In exploring the extent to which patient safety incident investigations are based on systems thinking, while effort and intent are evident, the execution fell short. A focus on person-level, individual factors and weaker recommendations revealed a gap between the systems approach and its application in practice. Incident investigations are limited by implicit and explicit constraints such as resourcing, investigator training and methodological challenges. Effective incident analysis requires five key areas of improvement, including: a need for sociotechnical incident analysis; improved data collection techniques; investigative independence; the professionalisation of incident investigators; and the aggregation of data. Integrating these improvements into incident analysis, as part of a system-wide proactive strategic response, can expand our understanding of the causes and recommendations to prevent further harm, ultimately improving healthcare. This is essential, especially in a healthcare system that is closely interlocked with social and cultural complexities which are becoming increasingly more complex as our population ages.

## Supplementary material

10.1136/bmjqs-2025-019063 online supplemental file 1

## Data Availability

Data are available upon reasonable request.
