# Supplementary material for: Do patient safety incident investigations align with systems thinking? An analysis of contributing factors and recommendations
Source: BMJ Qual Saf. 2025 Sep 12;35(7):e019063. doi: 10.1136/bmjqs-2025-019063 (PMC13311954; doi:10.1136/bmjqs-2025-019063)
Supplement: online supplemental file 1 [file bmjqs-35-7-s001.pdf]

SUPPLEMENTAL FIGURE – SAMPLING STRATEGY

Victoria

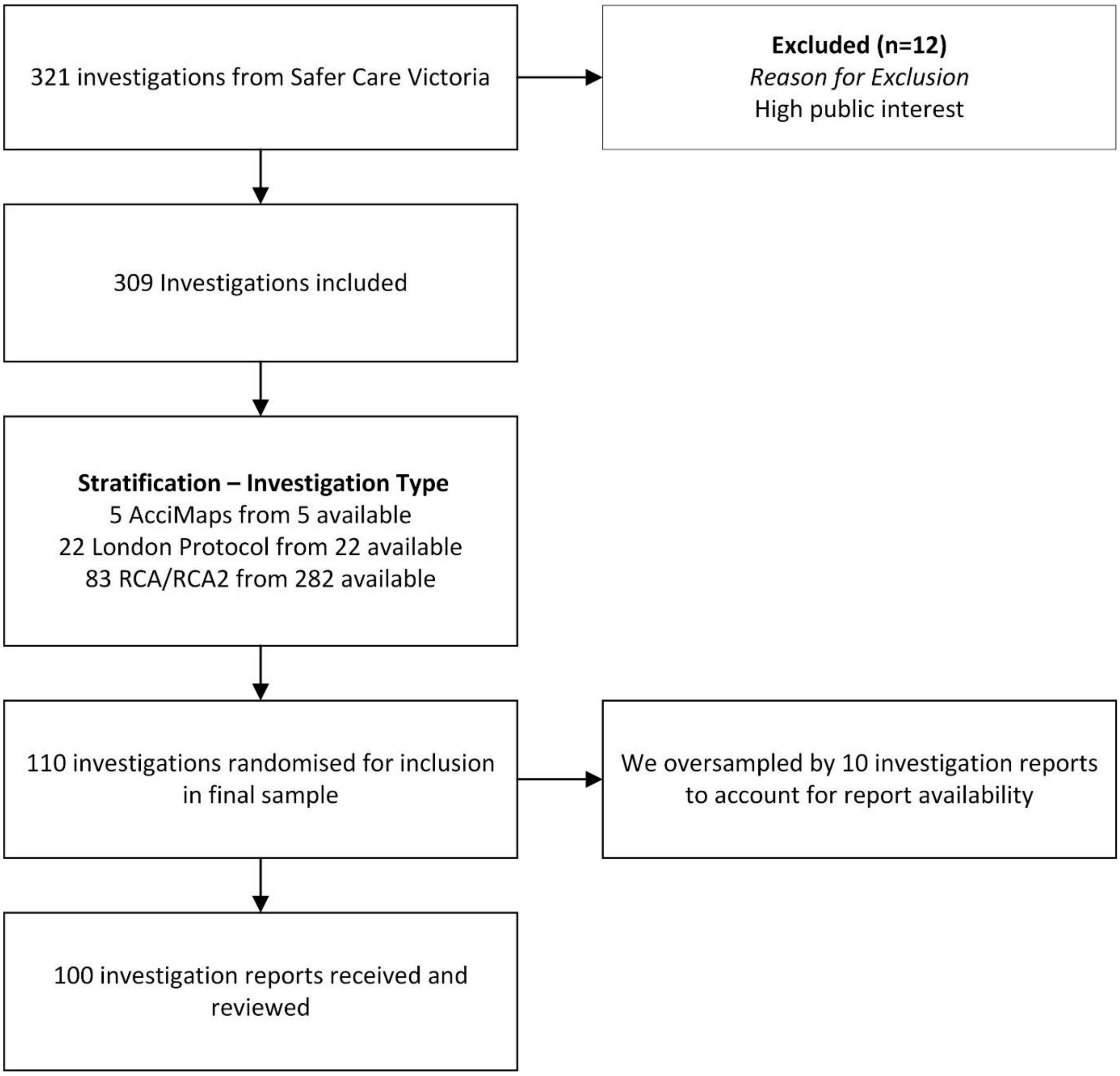

Queensland

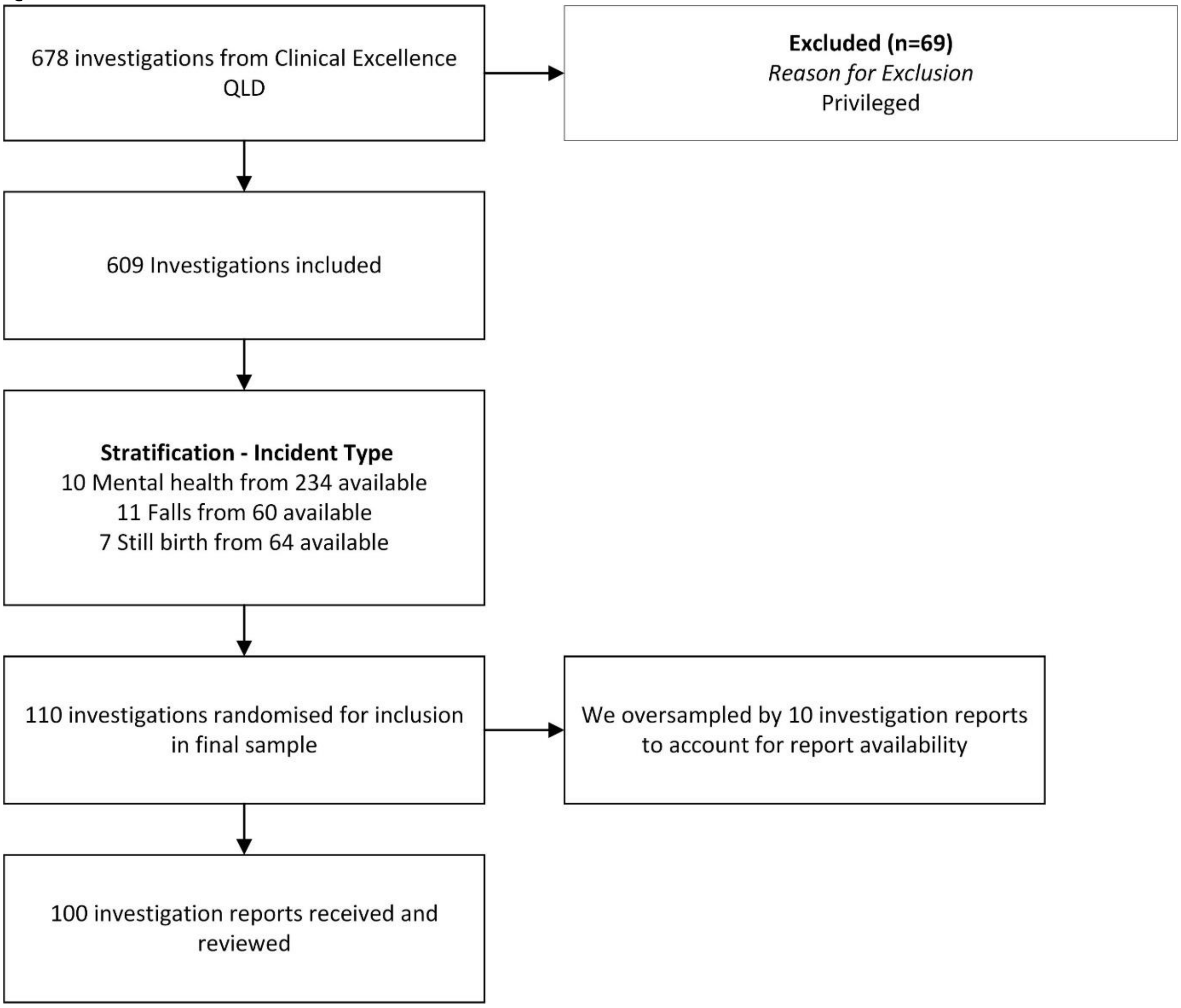

678 investigations from Clinical Excellence QLD
Excluded (n=69)
Reason for Exclusion
Privileged
609 Investigations included
Stratification - Incident Type
10 Mental health from 234 available
11 Falls from 60 available
7 Still birth from 64 available
110 investigations randomised for inclusion in final sample
We oversampled by 10 investigation reports to account for report availability
100 investigation reports received and reviewed

New South Wales

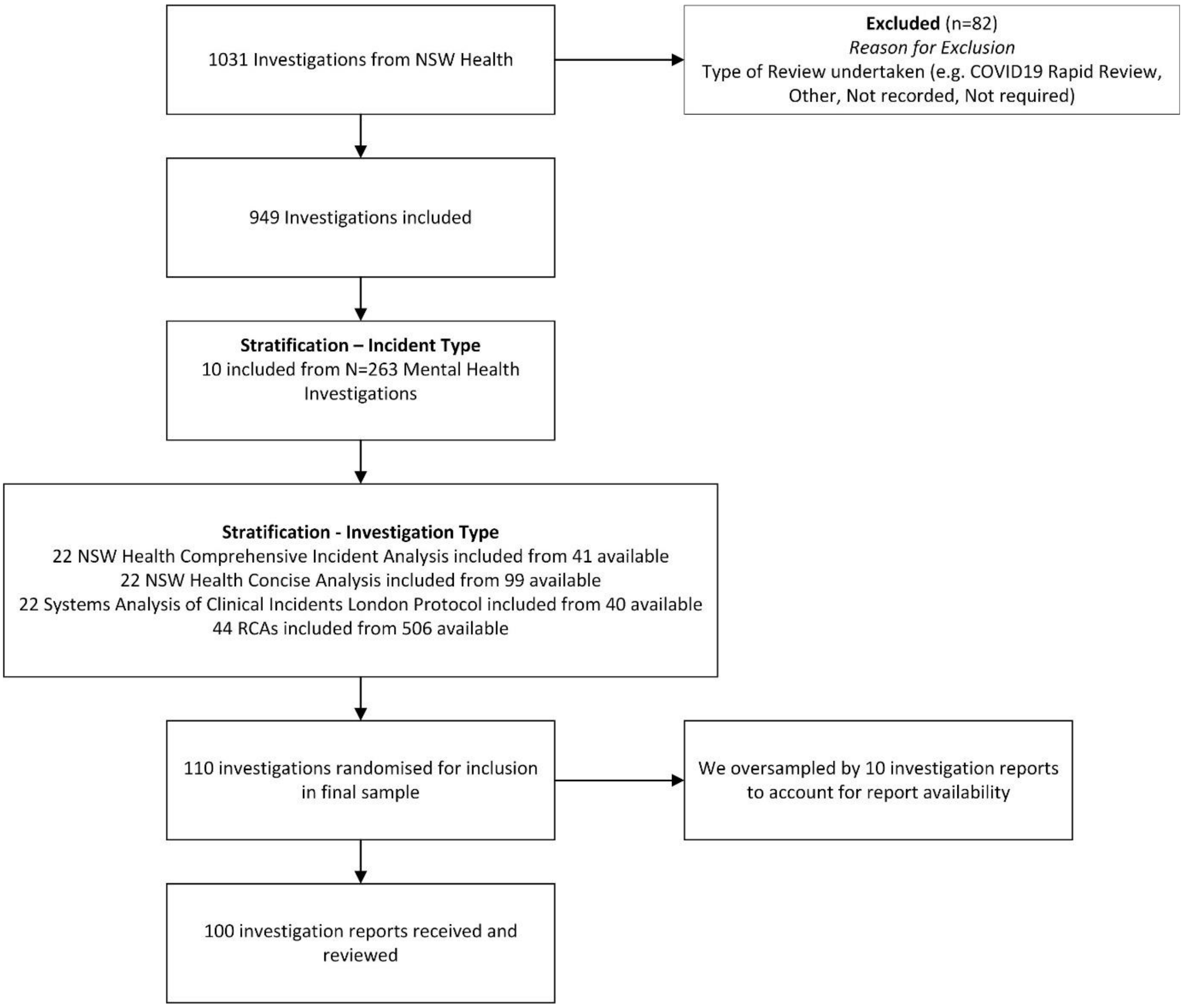

SUPPLEMENTAL TABLE – FRAMEWORK FOR THE CONTRIBUTING FACTOR CATEGORISATION

System level	System sub-level	Example
**External Environment**	Macro-Economic	Government budgets and funding structures
	Policy, Regulations & Targets	National and regional priorities for healthcare funding
	Societal Influences	Social and cultural influences
	Other	Environmental conditions
**Organisation**	Administrative Services	Electronic records
	Buildings & Facilities Management	Facilities management and maintenance
	Clinical/non-Clinical – Central Services	Pharmacy, Phlebotomy, Security, Catering
	Competency Management Systems	Learning and development
	Culture & Leadership	Organisational goals and incentives
	Policy & Procedures	Organisational directives and policies
	Strategy, Planning & Organisation	Strategic plans and directives
	Structure & Governance	Organisational structure and hierarchy
	Workforce Management	Staff scheduling, rosters, shift design
**Person**	Care Team	Communication, collaboration, cohesiveness
	Patient/Family	Clinical condition, complexity, disability
	Staff	Perception, attention, memory, knowledge, skill, decision-making
**Tasks**	Task Design	Task difficulty, complexity, ambiguity
	Workload	Job demand vs available capacity
**Tools & Technology**	Equipment & Devices	Ease of use of tools, forms, materials, technology
	Local Protocols & Job Aids	Local protocols and practices, diagnosis, or decision-making tools
**Internal Environment**	Workspace	Location, design, layout, noise
